# Low Diversity of Human Variation Despite Mostly Mild Functional Impact of De Novo Variants

**DOI:** 10.3389/fmolb.2021.635382

**Published:** 2021-03-18

**Authors:** Yannick Mahlich, Maximillian Miller, Zishuo Zeng, Yana Bromberg

**Affiliations:** ^1^ Department of Biochemistry and Microbiology, Rutgers University, New Brunswick, NJ, United States; ^2^ Department of Genetics, Rutgers University, Piscataway, NJ, United States

**Keywords:** variation, adaptation, evolution, nsSNVs, SNAP, cross-species variation, common variation

## Abstract

Non-synonymous Single Nucleotide Variants (nsSNVs), resulting in single amino acid variants (SAVs), are important drivers of evolutionary adaptation across the tree of life. Humans carry on average over 10,000 SAVs per individual genome, many of which likely have little to no impact on the function of the protein they affect. Experimental evidence for protein function changes as a result of SAVs remain sparse – a situation that can be somewhat alleviated by predicting their impact using computational methods. Here, we used SNAP to examine both *observed* and *in silico* generated human variation in a set of 1,265 proteins that are consistently found across a number of diverse species. The number of SAVs that are predicted to have any functional effect on these proteins is smaller than expected, suggesting sequence/function optimization over evolutionary timescales. Additionally, we find that only a few of the yet-unobserved SAVs could drastically change the function of these proteins, while nearly a quarter would have only a mild functional effect. We observed that variants common in the human population localized to less conserved protein positions and carried mild to moderate functional effects more frequently than rare variants. As expected, rare variants carried severe effects more frequently than common variants. In line with current assumptions, we demonstrated that the change of the human reference sequence amino acid to the reference of another species (a cross-species variant) is unlikely to significantly impact protein function. However, we also observed that many cross-species variants may be weakly non-neutral for the purposes of quick adaptation to environmental changes, but may not be identified as such by current state-of-the-art methodology.

## Introduction

The vast majority of human genomic variants are single nucleotide variants (SNVs) (Durbin, et al., 2010). Coding region variants trivially make up a much smaller fraction of all variation than do non-coding variants (Lander, et al., 2001). However, the former affect protein structure/function and thus have a disproportionate effect of molecular function of the cellular machinery. For example, each individual genome contains approximately ten thousand of nsSNVs (non-synonymous SNVs, which change the amino acid sequence (Shen, et al., 2013), a combination of which is responsible for a variety of observed phenotypes, including disease (Peterson, et al., 2013; Hassan, et al., 2019). Establishing the effect of any given nsSNV, however, is a difficult task. One gold-standard experimental approach is saturated mutagenesis (SM) (Wells, et al., 1985), which induces variants of interest in a gene and measures the change of resulting protein molecular function. However, SM is too inefficient to thoroughly study the entirety of genomic variation. While the recent development of the deep mutational scanning techniques (Fowler and Fields, 2014) has facilitated high-throughput functional analysis of coding variants, experimental annotation of millions of possible nsSNVs in human genome still remains elusive, Given the inefficiency of large-scale experimental measurements computational methods for variant effect interpretation offer a plausible alternative for the exploration of the human genome.

Genome-wide association study (GWAS) (Visscher, et al., 2017), as well as the *post hoc* polygenetic risk scoring (Torkamani, et al., 2018), has been extensively deployed to establish the associations between complex phenotypes and genetic background. GWAS results, however, are by definition association (not causation) evaluations and are specific to a phenotype. Evaluating variant effect on molecular function requires a different type of techniques. Machine learning models are often used to classify variants into neutral/deleterious (e.g., CADD (Kircher, et al., 2014), DANN (Quang, et al., 2014)), benign/pathogenic (e.g., MutPred2 (Pejaver, et al., 2017), PhD-SNP (Capriotti and Fariselli, 2017)), stable/unstable (e.g., I-Mutant2.0 (Capriotti, et al., 2005)), and effect/no-effect (e.g., Envision (Gray, et al., 2018), SNAP (Bromberg and Rost, 2007), SNAP2 (Hecht, et al., 2015)).

Conservation of residues across homologs is often assumed to indicate structural or functional importance of these residues and their intolerance to substitution (Kumar, et al., 2009). Thus, conservation is used as a proxy for variant effect evaluation, e.g. by tools like SIFT (Ng, 2003) and PROVEAN (Choi and Chan, 2015), and has been widely incorporated as one of the features in many other variant effect predictors (e.g., CADD, DANN, SNAP, PhD-SNP). We previously proposed the concept of cross-species variants (CSV) analysis (Mahlich, et al., 2017), which is similar to but intuitively different from conservation evaluation. Conservation can be directly computed from a multiple sequence alignment (MSA) of homologs built for CSV analysis. However, CSVs specifically describe only the difference between two orthologous reference sequences and do not summarize overall conservation. For example, if the amino acid residue at a specific position of a human protein is glycine, and if the MSA-corresponding position of a mouse ortholog is leucine, then a CSV at this position of this human protein would be glycine > leucine. If this particular glycine > leucine variant also occurs in the human population, the variant is an *observed* CSV. As a rule, these types of human variants, i.e. to residues found in other species, have been presumed to carry no effect on protein function (Ng and Henikoff, 2001; Ng, 2003; Calabrese, et al., 2009; Adzhubei, et al., 2010; Shihab, et al., 2013; Kircher, et al., 2014; Schwarz, et al., 2014; Pejaver, et al., 2020). After all, if an amino acid is observed in a functional protein of an ortholog, its substitution into the human version cannot be expected to drastically affect the function.

Pathogenic amino acid substitutions are, on average, functionally more radical than CSVs (Briscoe, et al., 2004; Miller and Kumar, 2001; Subramanian and Kumar, 2006). A study of the rhodopsin protein, for example, has revealed that variants corresponding to CSVs among vertebrates are less likely to be pathogenic (Briscoe, et al., 2004). Of the 7,293 human-mouse CSVs in 687 human disease genes, only a small fraction (2.2%) corresponds to known human disease variants (Waterston, et al., 2002). Other studies have also estimated that only about 10% of the human-to-other-species amino acid substitutions are involved in disease (Kondrashov, et al., 2002; Subramanian and Kumar, 2006). However, this type of logic may have precipitated a self-fulfilling prophecy, where CSVs that were annotated to be neutral in the development of variant effect-prediction methods (Bromberg and Rost, 2007; Adzhubei, et al., 2010; Kircher, et al., 2014; Pejaver, et al., 2017; Pejaver, et al., 2020) could bias the prediction of previously unseen CSV effects toward neutrality. While unlikely pathogenic, intuitively, a yeast version of the human protein may be less or more functionally efficient, may have unexpected structural effects given the rest of the protein sequence, or may participate in different/additional molecular pathways. Incorporating taxonomic distances between the species included in an alignment improves identification of variant effect (Malhis, et al., 2019). A deeper evaluation of CSVs in terms of their functional effects may thus be warranted.

We previously reported (Mahlich, et al., 2017) that amino acid CSVs have less predicted molecular functional effects on average than human variation recorded by the Exome Aggregation Consortium (Lek, et al., 2016). Here we extend this analysis, by investigating human variation in 1,265 proteins that have orthologs in 20 species spread across the eukaryotic branch of the tree of life. We evaluate the differences in functional impact of the variants that are observed within the human population against those not yet observed, but genetically possible. We show that common variants favor less conserved positions than rare variants, indicating a potential need for flexibility in sequence for the purposes of environment-driven adaptation. We also assessed the differences in predicted impacts on the function of human protein of cross-species variants (CSVs; variant amino acid is found in one of the 20 orthologs) and non-CSVs. We finally suggest that the lack of functional impact of CSVs might be overestimated by the current presumption that evolutionary persistence suggests functional neutrality.

## Methods

### Variant Collection

A total of 93,437 human protein-coding transcripts were extracted from GRCh37 p.13 assembly (Church, et al., 2011) in Ensembl BioMart (Kinsella, et al., 2011). From these, we selected 22,346 longest transcripts per gene. We removed transcripts from patches/alternate sequences (http://m.ensembl.org/info/genome/genebuild/haplotypes_patches.html), retaining 19,971 transcripts. For these, we artificially generated all possible non-synonymous single nucleotide variants (73,813,560 nsSNVs). We downloaded the Genome Aggregation Database (gnomAD v2, https://gnomad.broadinstitute.org/downloads) exome data (Karczewski, et al., 2020) and, using SAMtools (Li, et al., 2009), mapped the generated nsSNVs to the corresponding variant allele frequencies where available. We thus collected 2,951,998 variants with gnomAD allele count = 1 and 2,561,015 gnomAD variants with larger allele counts. The remaining 68,300,547 variants were not found in gnomaAD. Note that at the time of data collection gnomAD v2 was the most current version available. The current v3 version of gnomAD is only slightly different in relevant content as its reference genome, GRCh38, recapitulates 99% of GRCh37 (Pan, et al., 2019) and most differences between the two are in the non-coding regions, an area outside this study. We thus expect that results and conclusions reported here would not change with this update.

The allele counts of all nsSNVs causing the same single amino acid substitution (SAV) were further aggregated to represent the frequencies of individual SAVs (Eq. 1):

$$\mathrm{freq}\left(\mathrm{SAV}\right)=\frac{\sum_{i=1}^{k}n_{i}}{N},$$ (1)

where for any codon, $n_{1}$ … $n_{k}$ are counts of the specific SAV-causing alleles and N is the total numbers of sequenced alleles of that codon. Note that in the process of aggregation some *observed* (allele count >1) SAVs could be derived from the aggregation from multiple single allele nsSNVs. The aggregation of nsSNV frequencies into SAV frequencies, resulted in 2,564,652 *observed* (allele count >1), 2,918,355 *singletons* (allele count =1), and 60,601,329 *synthetic* SAVs in the 19,971 transcripts. Observed variants were further classified as *common* (*freq* (SAV) ≥ 0.01) and *rare* (*freq* (SAV) <0.01).

### Collection of Cross-Species Variants

Cross-species variants (CSVs) are the amino acid differences between the human reference protein sequence and the orthologous protein sequence of another species. For example, if the amino acid residue at the third position of the human protein sequence *P* is leucine, and if the amino acid residue at the same position in mouse orthologous protein sequence is glycine, then the CSV at this position in *P* would be L3G. Aiming to span the tree of life with species available in Ensembl BioMart (GRCh37), we considered 20 species for CSV analysis: yeast (*Saccharomyces cerevisiae*), worm (*Caenorhabdiis elegans*), fruitfly (*Drosophila melanogaster*), zebrafish (*Danio rerio*), xenopus (*Xenopus laevis*), anole lizard (*Anolis carolinensis*), chicken (*Gallus gallus*), platypus (*Ornithorhynchus anatinus*), opossum (*Monodelphis domestica*), dog (*Canis familiaris*), pig (*Sus scrofa*), dolphin (*Tursiops truncatus*), mouse (*Mus musculus*), rabbit (*Oryctolagus cuniculus*), tree shrew (*Tupaia belangeri*), tarsier (*Carlito syrichta*), gibbon (*Nomascus leucogenys*), gorilla (*Gorilla gorilla*), bonobo (*Pan paniscus*), and chimpanzee (*Pan troglodytes*). We identified the evolutionary distances of these species from *Homo sapiens* using the TimeTree database (Kumar, et al., 2017). All protein coding DNA sequences (CDS) of these 20 species were downloaded from the Ensembl database (Zerbino, et al., 2018) (release 94, https://uswest.ensembl.org/info/data/ftp/index.html). For every human protein coding transcript *T*, the available orthologous CDS for each of the 20 species was extracted using the Ensembl BioMart (Kinsella, et al., 2011). Each species may have multiple protein coding sequences orthologous to *T*, but only the longest one was selected. We performed multiple sequence alignment (MSA) of *T* and all its orthologs using PRANK (Löytynoja and Goldman, 2005), which translates CDS and aligns protein sequences. Of the 19,971 human transcripts in our set, 1,342 had a full set of the 20 species orthologs in the MSA. In these transcripts (940,328 amino acids) there were 183,540 *observed* (49,541 CSVs/133,999 non-CSVs), 228,774 *singleton* (52,550 CSVs/176,224 non-CSVs), and 5,118,164 *synthetic* SAVs (873,011 CSVs/4, 245,153 non-CSVs).

### Cross-Species Variant Effect Predictions

We generated SNAP (Bromberg and Rost, 2007) predictions for all variants in the 1,342 transcripts. SNAP predictions could be made for 1,265 of the proteins; a set of 77 sequences (832,697 variants) did not yield any predictions due to SNAP’s sequence length constraints (63 sequences), variant to sequence mapping errors (3 sequences), and unresolvable errors in the SNAP input feature extraction pipeline (11 sequences) as well as an additional 46,840 variants on the remaining proteins. Note that, as in all other proteins in our set, the vast majority (93%) of these variants were *synthetic* (4% *singleton* and 3% *observed*), suggesting that our analyses of effect trends should be largely unaffected by this missing subset. Thus, the final SNAP effect prediction dataset contained 4,650,941 variants in 791,040 positions among 1,265 proteins (Supplementary Table S1). Note that for this study we used the original SNAP tool instead of the more recent version SNAP2 (Hecht, et al., 2015). There were two reasons for this choice: 1) SNAP2 used OMIM (Amberger, et al., 2009) disease variants in training, a choice which does not directly reflect variant functional effects, and 2) SNAP effect prediction reliability scores strongly correlate with the functional effect strength (Bromberg, et al., 2013), an observation that has not been explicitly made for SNAP2.

### Variant Conservation Scores

For all residues of all proteins in our set we computed two types of conservation scores:

1. We used the PredictProtein pipeline (Yachdav, et al., 2014) to compute ConSurf (Glaser, et al., 2003) conservation scores. ConSurf scores are based on MSAs of up to 150 homologous sequences. Reported scores are normalized so that the average score over all residues of one protein is zero and the standard deviation is one. Lower scores indicate more conserved residues.
2. We extracted from the list of SNAP input features the position-specific independent counts (PSIC) (Sunyaev, et al., 1999). PSIC scores reflect per-residue position-specific weights considering the MSA-based overall level of sequence similarity.

We only retained the conservation scores for those variant positions (104,375) that had both ConSurf and PSIC annotations.

Conservation scores across variant subsets were used only once per variant position in the subset. That is, if two rare CSVs were present at one protein position, conservation for this position was only used once toward establishing the distribution of the rare CSV dataset. On the other hand, if a position contained both a common CSV and a rare CSV, the conservation score was included separately into distributions of each subset.

### Per-Residue Funtrp Scores

funtrp (Miller, et al., 2019) is a prediction tool that assesses the expected range of functional effects due to the possible variants at a given protein position. funtrp classifies sequence positions as *neutral* (most variants at this position show weak or no effect), *rheostatic* (a full range of variant effects) and *toggle* (most variants have a severe effect). funtrp was trained with deep-mutagenesis data and uses sequence-based features to differentiate between the three residue classes. We used our publicly available webservice (https://services.bromberglab.org/funtrp) to identify funtrp classes for each position of 1,254 of our protein sequences; predictions for the remaining 11 sequences were not returned by the method.

### Evaluating Statistical Significance Distribution Differences

For all comparisons of score distributions (e.g. SNAP scores) across variant classes (e.g. rare vs common), we re-sampled said distributions 1,000 times to extract 1,000 observations each time. For each resampling instance, we performed the Kolmogorov-Smirnov test to test the equity of the distributions, reporting the associated *p*-value; the median p-val over 1,000 iterations was reported.

## Results and Discussion

### Many Variants Remain to be Sequenced

Single amino acid variant (SAV) effects were determined by SNAP (Bromberg and Rost, 2007) (predicted score range for our variants [−94, + 88]), with negative scores identifying neutral SAVs (no change in function) and positive scores identifying non-neutrals/effect SAVs (activating or deactivating changes in function); score absolute values indicate the reliability of prediction and, for non-neutral variants, the size of the effect (Bromberg, et al., 2013). Note that our definition of effect does not specify whether the effect is detrimental or beneficial to the organism, but rather reports on the change in wild-type functionality of the affected protein.

**FIGURE 1:**
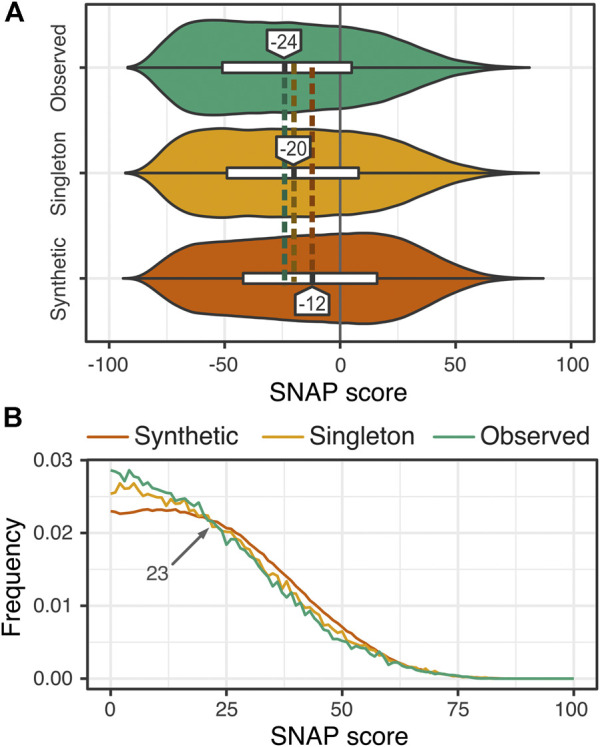
**Higher prevalence of effect among the synthetic as compared to observed and singleton variants**. **(A)** The distribution of effect predictions for synthetic variants (dark orange; median SNAP = -12) is significantly more right-shifted toward effect (SNAP ≥0; horizontal line) than that of observed variants (green; median SNAP = -24) and singletons (yellow; median SNAP = -20). For all distributions, however, the majority of predictions are neutral (SNAP <0) **(B)** Additionally, synthetic variants show an enrichment of moderate to severe functional effects (SNAP ≥ 23) vs. singletons and observed variants.

Overall, more variants were predicted to be neutral than effect, with some difference in fractions of effect variants between *synthetic*, *singleton*, and *observed* variant subsets (Supplementary Table S1). The distribution of synthetic variant SNAP scores was significantly different from that of singleton and observed variant scores (Kolmogorov-Smirnov, KS, test *p*-value; synthetic vs. singleton = 8.7*e*−04, synthetic vs. observed = 1.1e−06), while singleton and observed scores were only slightly different (singleton vs. observed *p*-val = 0.14). For *synthetic* variants (median SNAP score = −12; Figure 1A), i.e. those that have not been seen in the population, the majority (60%) were predicted to be neutral. These variants are, thus, technically *observable* and may be identified in future sequencing efforts. Those 40% of the synthetic variants predicted to have an effect, had on average more severe impact than the effect variants seen in the human population (combined *observed* and *singleton* sets; 31% effect; Figure 1B). Increased predicted effect of synthetic variants is in line with the expectation that these are subject to purifying selection.

Earlier (Bromberg, et al., 2013), we observed a similar trend of more effect variants in the *synthetic* than in the *observed*/*singleton* set; i.e. 55% effect in *synthetic* SAVs in 100 randomly selected enzymes vs. 46% effect variants in 1000Genomes data (Auton, et al., 2015). However, the fractions of both the *synthetic* and *observed*/*singleton* of effect variants in our earlier study were significantly higher than the corresponding numbers reported here. Furthermore, the SNAP scores of the *synthetic* variants reported here and those in Bromberg were significantly different (*p*-val 4.0*e*−15); the scores of our combined *observed/singleton* variants also differed from the scores of 1000Genomes variants (*p*-val = 3.0e−12).

While 1000Genomes variants were observed in 85% (1,072 of 1,265) of the transcripts used in this study, our variant set for these proteins was larger, suggesting improved sequencing coverage and accounting for some effect prediction differences. Notably, only 36% of the 1000Genome variants in our proteins had an effect–in line with the 31% effect variants in our *observed/singleton* set and 10% less than in the complete 1000Genomes variant set. Furthermore, of the set of 100 enzymes used in the Bromberg et al. study to generate synthetic variants, only four were present in our protein set. Thus, the difference in effect scores between our earlier study and the current work is most likely due to the specific genes/proteins selected for this study. Genes/proteins in our set have orthologs in each of our selected species, i.e. these are likely ancient and rarely disease-associated (Moreau and Tranchevent, 2012). As the functions of these proteins are important for organism survival, they likely harbor the variants necessary for environment-driven functional adaptation but do not allow for severe disruption upon mutation (Key, et al., 2014; Key, et al., 2014; Ilardo and Nielsen, 2018; Rees, et al., 2020). While the variants in these proteins may still be extremely deleterious, less than three percent in our set were of severe effect (SNAP score ≥50; 130,870 variants; 7.2% of all effect variants) and, as expected, most were *synthetic* (123,962 variants, 3% of all *synthetic*), with few found in the population (6,908 variants, 2% of all *singleton*/*observed*).

Given these fractions of effect variants, we expect at least half a million (neutral *synthetic* CSVs) and possibly over four million (any *synthetic* neutrals and milds/moderates) variants to be possibly observable, i.e. they may be found with more sequencing. As the genes considered here are likely ancient and evolutionarily optimized to resist drastic changes upon mutation, this 12-fold possible increase in the observable variants (vs those already observed) suggests an upper bound of increase in the number of *observed*/*singleton* SAVs that may be collected in the future.

### Common Variants May Drive Environmental Adaptations

Despite the fact that common variation is, by definition, widespread in the population, trivially, the vast majority of unique population variants are rare. Variant effect trends are therefore dominated by observations for rare variants, effectively drowning out signal from common variants. We thus aimed to elucidate the difference between common (≥1% SAV frequency) and rare variants. For this part of the analysis we excluded from consideration the *singleton* variants, which are a special case of rare variation and may be disproportionately sequencing errors. We note that common variants are unlikely to be very deleterious/disease-causing as they would not stay common. On the other hand, variants that have no impact on function (*neutrals*) and very weak nonneutrals can be fixed in the population at about the same rate via genetic drift (Kimura and Ohta, 1969).

We also considered the differences between *observed* cross-species variants (CSVs) and non-CSVs (Methods). We expected different evolutionary drivers for the existence of different variant types (e.g. common CSV vs. rare non-CSV) and, in turn, potential differences in their impact on protein function. Note that variants labeled as non-CSV may still be present in the orthologs of species that were not assessed here. However, using more species could also reduce our total protein set if some of the currently used transcripts are absent in the new species transcriptome.

Common variants are as frequently CSVs as non-CSVs (691 CSVs vs. 683 non-CSVs, Supplementary Table S2). For common CSVs (*reference* substituted by *variant* amino acid), the human reference amino acid is present in a minority (40%) of all 20 species orthologs, but more frequently in mammals (48%) and great apes (59%; Table 1). Note that these fractions were computed as the number of shared reference amino acids of all residues aligned, e.g. if for one variant ten of 15 orthologs aligned at the variant position have the human reference amino acid, while for another variant four of the 20 orthologs do, the total fraction of reference amino acid across these variants is 40% (14/35). Given these fairly low fractions, the *variant* amino acids of common CSVs are possibly ancestral, i.e. human *variant* amino acid could have been the *reference* of a potential ancestor. Thus, for humans reinstating the ancestral residue at this position is likely to be detrimental, as it would otherwise remain fixed as reference.

**TABLE 1:** Prevalence of human reference amino acids in CSV positions across orthologs.

	Rare		Common	
	CSV (%)	Non-CSV (%)	CSV (%)	Non-CSV (%)
Apes	98	99	59	98
Mammals	83	93	48	86
All	68	83	40	75

For common non-CSVs the corresponding fractions of reference amino acids across orthologs are 75% (all), 86% (mammals), and 98% (apes; Table 1). Thus, *variant* amino acids of common non-CSVs likely represent somewhat newer evolutionary developments and are 1) likely to be beneficial (still effect!) for humans as a whole but may have not been around long enough to become the reference or 2) are non-universal adaptations to persistent environmental conditions, e.g. ethnicity-specific variants (Rees, et al., 2020).

Unlike common variants, rare CSV variants are nearly three-fold less commonplace than non-CSVs. However, just as for common variants, rare non-CSV *reference* amino acids are present in orthologs at a higher frequency than CSV references (83% non-CSV vs. 68% CSV). The preponderance of non-CSV reference amino acids across all species highlights these variants as likely of recent origin, and therefore possibly of any amount (a full range) of effect. Rare CSV variant amino acids, on the other hand, may be ancestral, although the likelihood of this is greatly diminished as compared to common variants (68% rare vs. 40% common reference amino acid across orthologs). If they are ancestral, their extensive elimination from the population would suggest deleterious effects (purifying selection). Independent appearances of the variant in human (as rare variant) and in another species (as reference) is unlikely, but also possible. In this case, the variant amino acid would likely be neutral or slightly deleterious in human.

**FIGURE 2:**
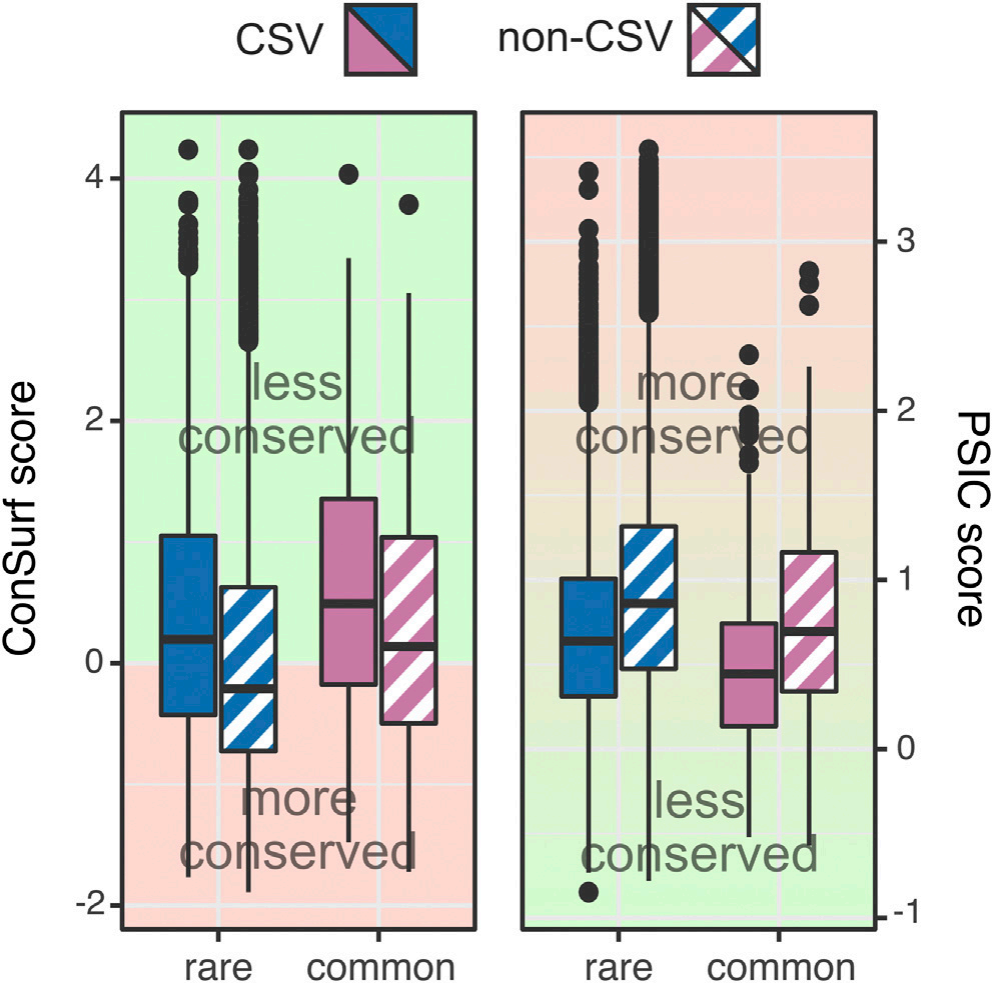
**Rare variants more frequently found in conserved protein positions**. Rare variants (blue) are more frequently found in conserved positions (ConSurf ≤0) than common variants (purple). Furthermore, non-CSVs (hatched fill) are more frequently present in conserved positions than CSVs (solid fill). Similarly, rare variants carry higher PSIC scores than common variants.

Further comparing the frequencies of occurrence of reference amino acids across orthologs suggests that rare variants occur at more conserved positions than common variants; reference amino acids of CSVs *vs.* non-CSVs were present across all species for 68% vs. 83% for rare variants and 40% vs. 75% for common ones. Evaluation of conservation of variant positions using ConSurf (Glaser, et al., 2003) confirmed this observation (Figure 2; lower score means more conserved position; KS p-val CSV rare vs. common = 3.2*e*−08, non-CSV rare vs. common = 1.5*e*−09). The protein positions harboring rare variants were on average more conserved (103,609 positions; median ConSurf score = −0.11) than positions with common variants (1,013 positions; median ConSurf score = 0.36). Note that there are only a few 247) positions for which both rare and common variants are present, and these are also only weakly conserved (median ConSurf score = 0.34). A similar trend was observed using PSIC scores (Sunyaev, et al., 1999) of variant positions (Figure 2; higher score means more conserved position; median PSIC score of: rare = 0.80, common = 0.55, both = 0.59; KS *p*-val CSV rare vs. common = 4.4*e*−16, non-CSV rare vs. common = 4.2*e*−07).

**FIGURE 3:**
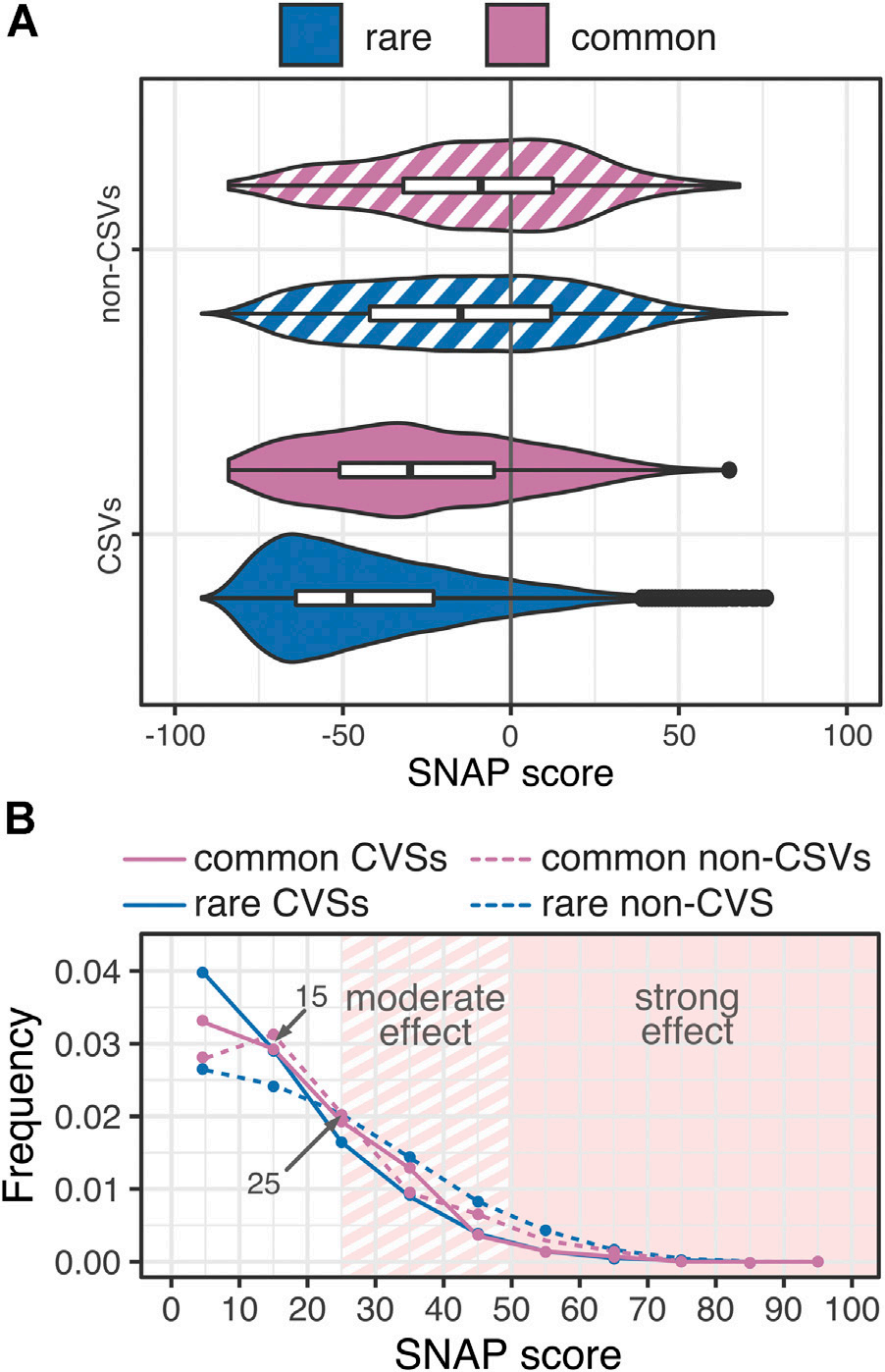
**Common variants are more frequently effect than rare variants, but rare variants are more frequently severe**. **(A)** The distribution of common CSV and non-CSV predictions (purple) is more right-shifted (more effect) than that of rare variants (blue). Furthermore, **(B)** rare non-CSVs (blue dashed line) are more often of moderate and severe effect than common non-CSVs (purple dashed line). However, common CSVs are more often of mild-moderate effect than rare CSVs. Due to small numbers of variants at each SNAP score (*x*-axis), frequencies are calculated in intervals of 10, e.g. 0 ≤ SNAP <10; points are centered in the interval.

This is an unexpected result, as variants in conserved positions are often assumed to have an effect, while rare variants, both CSV and non-CSV, are less frequently predicted to have an effect than the corresponding common variants (rare *vs*. common effect variants: 10% *vs.* 20% CSVs and 36% *vs*. 40% non-CSVs; Supplementary Table S2). Here we point out that more severe effect (several high score outliers) vs. more frequent effects (many variants have some effect) indicate different score distributions but may result in similar summary statistics (e.g. distribution means). Thus, although common variants have an effect more frequently than rare variants (Figure 3A), the former are less frequently severe (SNAP ≥50; 6% rare vs 3.6% common effect variants; Figure 3B). Furthermore, rare non-CSVs are enriched in moderate effect variants (SNAP ≥25) vs. common non-CSVs that are mostly mild. Common CSVs, on the other hand, carry more moderate effects than rare CSVs (Figure 3B). Note that as CSVs in general score tend to be predicted neutral more often than non-CSVs (Supplementary Figure S1, the preponderance of high-scoring common CSVs vs. non-CSVs reinforces the likely adaptational value of common CSVs proposed above. The propensity of rare variants to cause severe effects highlights them as likely culprits of disease. However, rare variants make up nearly three quarters of variation overall and are clearly not restricted to being disease-causing. In fact, they cover a complete range of effect–from strongly effect to reliably neutral (Figure 3A).

**FIGURE 4:**
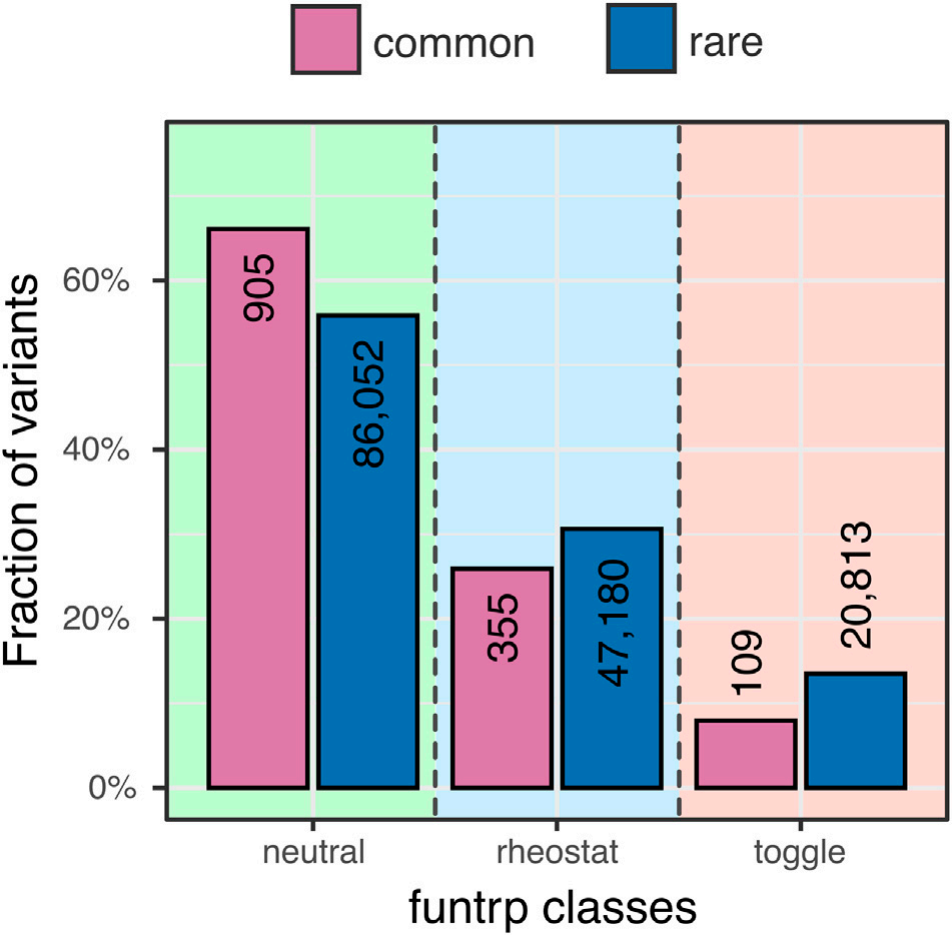
**Common variants prefer neutral positions more than rare variants**. Neutral positions (green shading) are enriched in common variants (purple) as opposed to rare variants (blue) (66% vs. 56% - actual variant counts shown as numbers in the bars). The fraction of rare variants in rheostatic positions (blue shading) is higher than the corresponding fraction of common variants. However, the ratio of common variants in rheostat positions vs. toggles (pink shading) is higher than that of rare variants.

In an effort to validate our observations of effects of common variants we used funtrp (Miller, et al., 2019) – a method that trained to recognize the range of variant effects possible at a single protein position. It classifies positions into 1) neutrals, where most variants have no effect on protein function, 2) toggles, where most variants have severe or knockout effects, and 3) rheostats, where variants cover a range of effect strengths. Overall, funtrp classes reflected SNAP predictions well; median SNAP scores of variants in neutral, rheostat, and toggle positions were −33, −18, and 12, respectively. Common variants were more often found in neutral positions as compared to rare ones (66% vs. 56%, Figure 4A). However, of the effect positions (i.e. rheostat and toggle), common variants preferred rheostats (77% common vs. 69% rare variants). As most toggles are conserved (Miller, et al., 2019), this observation is in line with the above finding that rare variants 1) are more likely than common ones to be in conserved positions and 2) that they carry more severe functional effects. Common variants in rheostatic positions, on the other hand, were likely used in evolution to fine-tune functions of affected proteins.

### Variant Effect Reflects Evolutionary Time of Reference Amino Acid Origin

**FIGURE 5:**
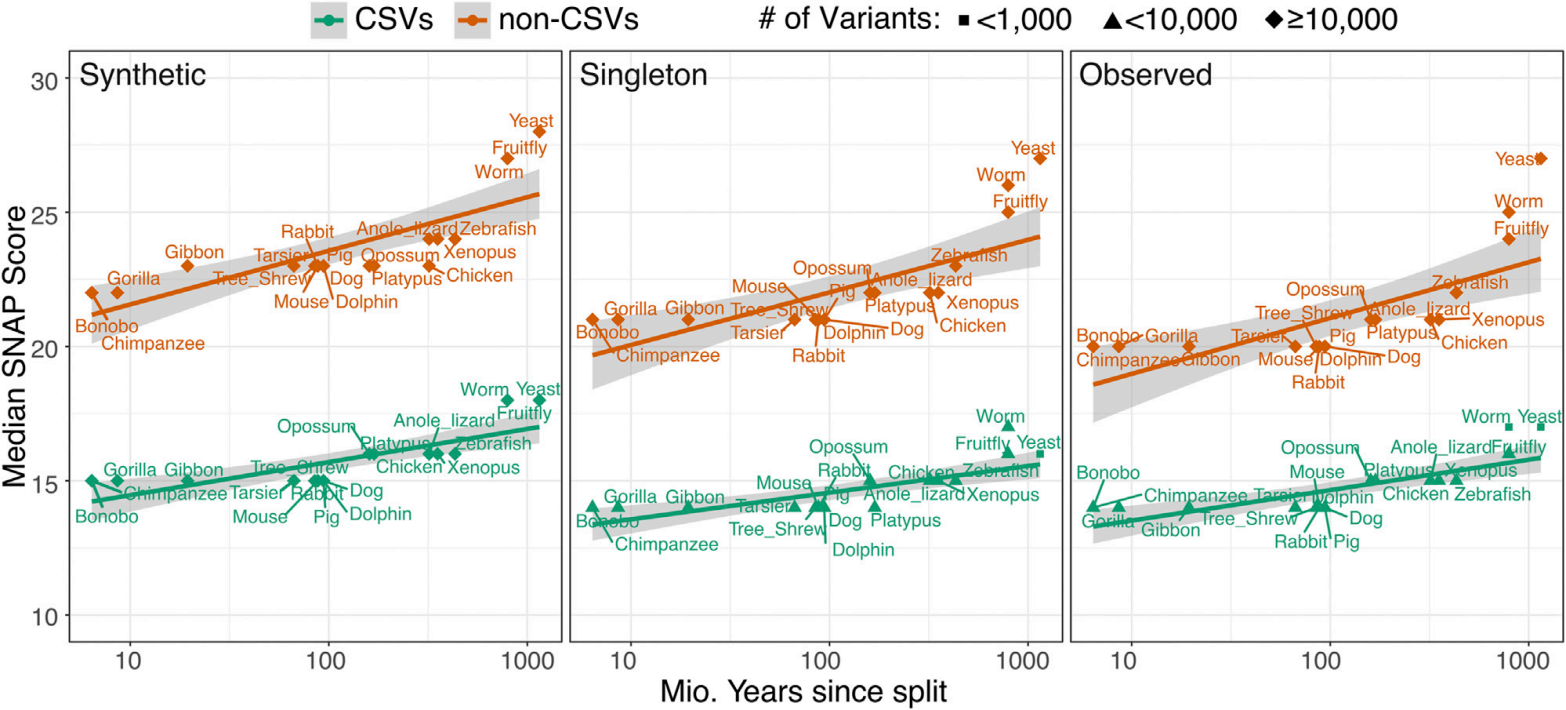
**Impact of variants sharing reference amino acids with other species correlates with evolutionary distance.** Mean SNAP scores (y‐axis) are computed for CSV (green line) and non‐CSV (red line) synthetic (left panel), singleton (middle panel), and common (right panel) variants, according to per-species human-shared reference amino acids. Species are placed along the x‐axis (logarithmic) according to their distance to ancestor shared with human.

We asked whether variant effect is related to the likely evolutionary time of appearance of the human reference. For each species *X*, we collected all effect variants in our dataset where the human and *X* reference amino acids were identical. For mammals, the median effect strengths of the variants affecting these positions were similar. For other species, however, the variant effect was correlated with increasing evolutionary distance between human and the specific species (Figure 5). This correlation held true for CSVs and non-CSVs, as well as for *synthetic*, *singleton* or *observed* variants.

Notably for non-CSVs, median effect scores increased more rapidly over evolutionary time than for CSVs. This trend was expected, as variants whose reference amino acids are present in evolutionarily distant species likely disproportionately affect conserved ancestral amino acids. For example, a shared human and yeast reference amino acid is likely present across all or most species in our set. Thus, a CSV at this position (if say, fly amino acid is different) would indicate some flexibility at the position, but a non-CSV would elicit the functional effect associated with the disruption of stringent conservation. However, we found that conservation of the variant position is unlikely the sole contributor to the observed effect gradient. The trend, albeit less pronounced, remained visible if only the variants in positions of low conservation (ConSurf score ≥0.5) were used in the analysis (Supplementary Table S2). Importantly, a clear distinction between CSVs and non-CSVs was also still evident, indicating that even in non-conserved positions CSVs and non-CSVs are distinguishable.

### Self-Fulfilling Prophecy: Are Cross-Species Variants Really Neutral?

**FIGURE 6:**
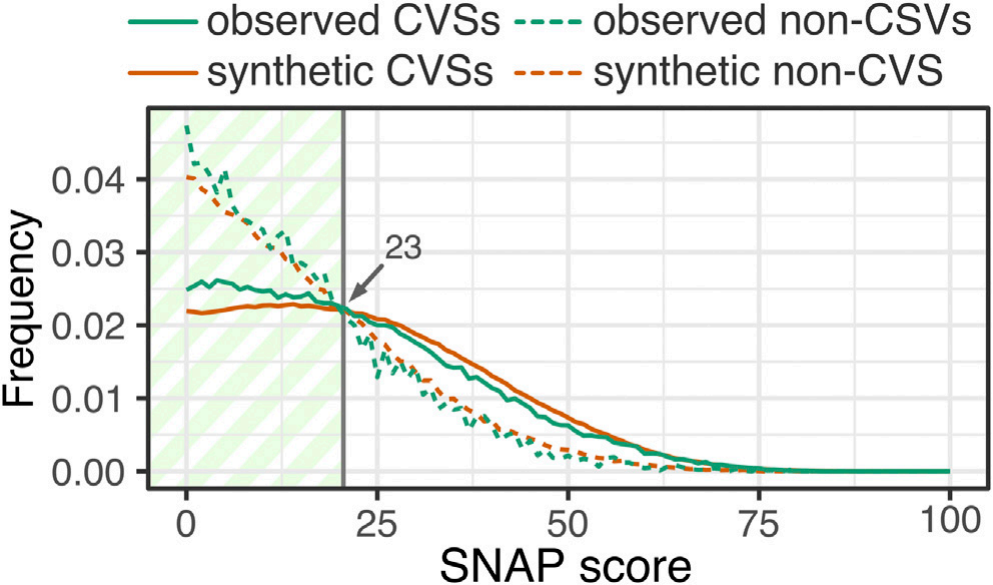
**Observed variants enriched in mild effects**. Both observed CSVs (green solid line) as well as non-CSVs (green dashed line) are enriched in mild effect variants over their synthetic counterparts (orange CSVs–solid line, non-CSVs–dashed line).

As mentioned previously, CSVs were less often predicted to have an effect than mutations to an amino acid that is not present in other species (non-CSVs); this observation was true for both *synthetic* and observed human variation (Supplementary Table S1). The absolute difference in median SNAP scores between CSVs and non-CSVs was 38 (mean =30) for *synthetic* variants and 32 (mean =27) for the *observed*–a full 14–21% of the entire scoring range ([−94, + 88]). CSV scores are most often neutral across all three categories of variation (i.e. *synthetic*, *singleton*, *observed*), while the distribution of non-CSV scores is much more widespread (Figure 6). An biological explanation for this observation is that CSVs are indeed more likely to be neutral with respect to protein function, as is expected from their persistence in homologs (Kondrashov, 1995; Sunyaev, et al., 2001). However, another explanation for this stark difference could then be the fact that SNAP was trained using a dataset of cross-species orthologous enzyme variants deemed neutral. Only 30 of these enzymes were in our set of 1,265 proteins and, thus, are not expected to dramatically impact our observations. However, if SNAP learned input feature patterns specific to CSVs, others could be labeled neutral without ever being seen in training. Thus, SNAP could fail to recognize CSVs that have a functional impact without introducing the organism to selection pressures, i.e. functionally non-neutral, but physiologically neutral. In fact, these may be the so called “fuel for evolution” (Bromberg, et al., 2013; Fu, et al., 2013) – the pool of weakly nonneutral variants necessarily present in the population for the purposes of quick adaptation to a changing environment.

In our earlier work we had determined a SNAP threshold of 23 as the upper functional impact limit to the absence of physiological visibility. We have confirmed this threshold for this data set as well, as the score where the fraction of possible/expected variants exceeds those observed (Figure 6). Of the *observed* effect CSVs, 76% are in this mild functional effect range, while 58% of all effect non-CSVs are as well. This significantly larger fraction of mild effect CSVs than effect non-CSVs suggests that the former are more likely the functional variants necessary for adaptation.

Although CSVs are more frequently (vs non-CSVs) predicted to be mild in effect, they also vastly outnumber non-CSVs in the neutral score range. Curiously, there is almost no difference between the *synthetic* and *observed* CSV score distributions. However, only 5% of all possible CSVs in our set are observed in the human population–not much more (percentage-wise) than all possible non-CSVs (3%; and fewer in the absolute sense with ∼42K *observed* CSVs and ∼144K *observed* non-CSVs). It thus remains unclear whether functional constraints are indeed weaker for (often biochemically similar substitutions of amino acids in) CSVs.

Evaluating prediction bias is difficult in the absence of a gold-standard data set and one of neutral CSVs doesn’t exist. While funtrp uses site conservation as input, it was not trained to recognize individual variant effect and thus could be used to elucidate our findings. In other words, funtrp forgoes the broad generalization of assigning neutrality to cross-species variants on the basis of the evolution-guided inference (e.g. SNAP and other methods (Ng and Henikoff, 2001; Ng, 2003; Calabrese, et al., 2009; Adzhubei, et al., 2010; Shihab, et al., 2013; Kircher, et al., 2014; Schwarz, et al., 2014; Pejaver, et al., 2020).

In line with our earlier observations, funtrp found that most protein positions in our set are neutral. The distribution of *synthetic*, *singleton*, and *observed* variants across position classes was very similar for CSVs (62/30/8% neutral/rheostat/toggle; Supplementary Table S3). Non-CSVs maintained an average 50/33/17% ratio of neutrals/rheostats/toggles, with *observed* non-CSVs more frequently found in neutral and rheostat positions than *singletons* or *synthetic* variants (Supplementary Table S3). Thus, both CSVs and non-CSVs were about as likely to localize to rheostatic positions, but non-CSVs were less frequently found in neutrals and twice as often in toggles. Note that while not all variants in neutral positions are necessarily functionally neutral, and non-neutral positions may have some neutral variants, only 62% of observed CSVs are found in neutral positions, while SNAP predicts 90% of observed CSVs to be functionally neutral.

Two conclusions from these results are salient: 1) as expected, CSVs are indeed more frequently neutral than non-CSVs and 2) it appears that SNAP (and likely other predictors) tends to overestimate CSV neutrality. Thus, we suggest that cross-species variants may carry mild to moderate functional effects and should be evaluated accordingly.

## Conclusion

We investigated a set of single amino acid substitutions (SAVs) in evolutionarily persistent, likely ancient, proteins, i.e. those that we expect to be optimized to tolerate variation. We found that despite the enrichment in severe effects of *synthetic* vs *observed* variants, a large proportion of SAVs might still be found upon broader sequencing of the population. Moreover, we expect that only a small fraction of variants that have yet to be sequenced will have a severe impact and/or be disease causing. We further observed that common variants favor poorly conserved sites. This lower conservation, indicative of more tolerance toward variation, might be providing enough “wiggle” room for environmental adaptations. Rare variants are, on the other hand, are often found in more conserved positions, explaining their enrichment in severe effects in comparison to common SAVs. Curiously, it appears that our ancient proteins have been optimized to the point where disrupting a conserved site does not immediately cause a functional disruption, as seen in the majority of rare variants predicted to be neutral. Finally, we suggest that cross-species variants (CSVs) might indeed be more often neutral than non-CSVs however not as consistently as currently expected. Ultimately, however, this question can only be answered through the development of an effect predictor that is does not make a priori assumptions of CSV neutrality and, which is somewhat harder, does not rely on conservation.

## Data Availability

The original contributions presented in the study are included in the article/Supplementary Material, further inquiries can be directed to the corresponding author.
