# Supplementary material for: Low Diversity of Human Variation Despite Mostly Mild Functional Impact of De Novo Variants
Source: Front Mol Biosci. 2021 Mar 18;8:635382. doi: 10.3389/fmolb.2021.635382 (PMC8012514; doi:10.3389/fmolb.2021.635382)
Supplement: Supplementary file 3 [file table1.docx]

Supplementary Material

# Supplementary Data

**Supplementary Data 1.** CSV containing human SAV frequencies and cross-species amino acid information

**Supplementary Data 2.** CSV containing effect predictions for human SAVs including SAV frequencies

# Supplementary Figures


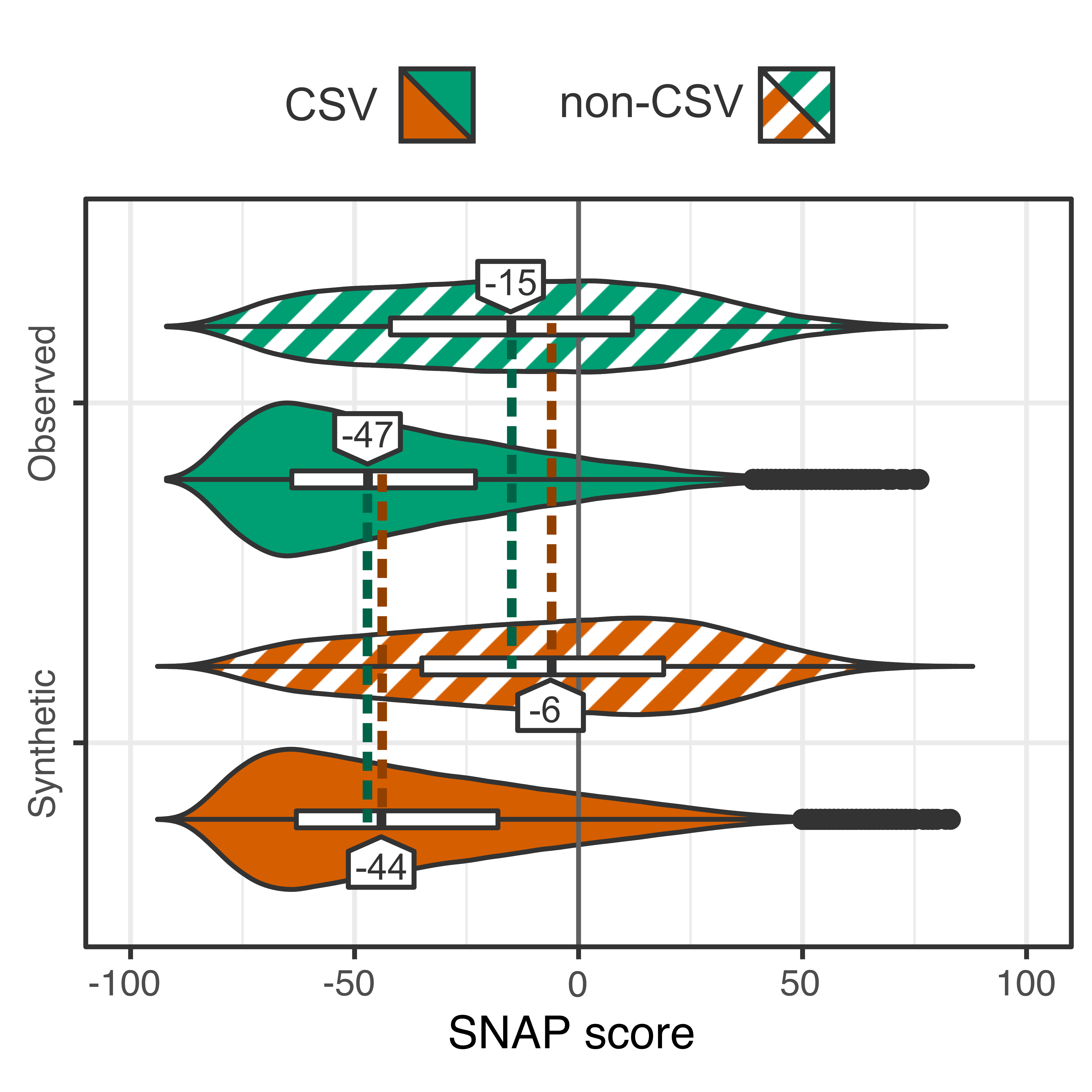


**Supplementary Figure 1. Large difference between cross-species (CSV) and non-cross-species (non-CSV) variants.** There is a larger difference in effect predictions between CSVs (solid) and non-CSVs (hatched) variation than between observed (green) and synthetic (dark orange) variants. CSVs are overwhelmingly predicted to be neutral (observed CSV median SNAP = -47, synthetic CSV median SNAP = -44).


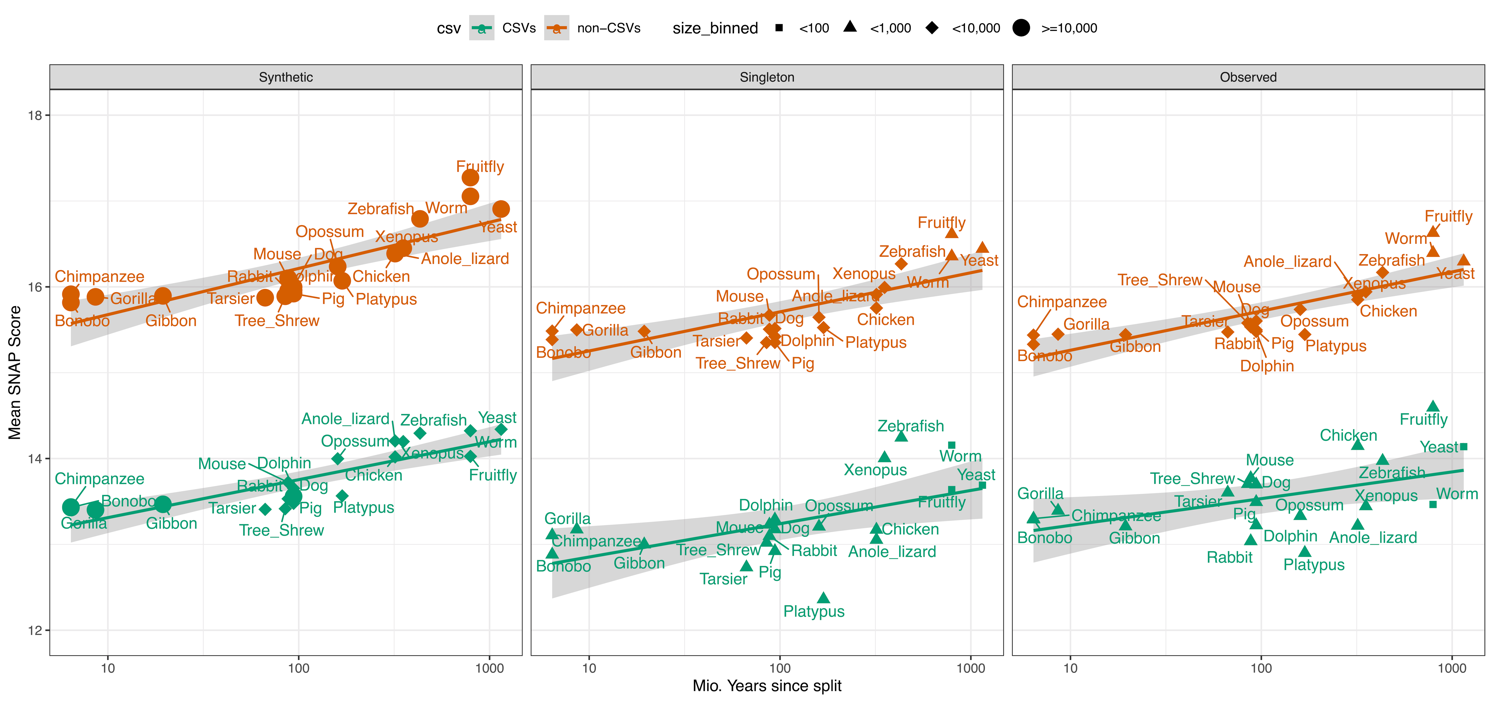


**Supplementary Figure 2. Impact of variants sharing reference amino acids with other species in less conserved positions (ConSurf ≥0.5) correlates with evolutionary distance.** Mean SNAP scores (y-axis) are computed for CSV (**green** line) and non-CSV (**red** line) *synthetic* (left panel), *singleton* (middle panel), and *common* (right panel) variants, according to per-species human-shared reference amino acids. Species are placed according to their age since speciation event (x-axis – logarithmic).

# Supplementary Tables

**Supplementary Table 1. Distribution of predicted effects across variant subsets.** Column of ‘Neutral’ and ‘Effect’ list variant fractions of ‘All’ (combined neutral & effect) Column. ‘All’ column list variant fractions of ‘Total’ by CSV / non-CSV.

|  | Neutral  *Count (% of all) \|*  *Median/Mean* | Effect  *Count (% of all) \|*  *Median/Mean* | All  *Count (% of total) \|*  *Median/Mean* |
| --- | --- | --- | --- |
| Synthetic  CSV  Non-CSV |  | | |
|  | 640,674 (87%) \|  -50 / -47 | 98,222 (13%) \|  14 / 17 | 738,896 (89%) \|  -44 / -38 |
|  | 1,954,065 (55%) \|  -31 / -34 | 1,608,290 (45%) \|  22 / 24 | 3,562,355 (93%) \|  -6 / -8 |
| Singleton  CSV  Non-CSV |  | | |
|  | 39,590 (89%) \|  -51 / -48 | 4,877 (11%) \|  13 / 16 | 44,467 (5%) \|  -47 / -41 |
|  | 90,826 (61%) \|  -34 / -35 | 57,928 (39%) \|  20 / 23 | 148,754 (4%) \|  -12 / -13 |
| Observed  CSV  Non-CSV |  | | |
|  | 37,961 (90%) \|  -51 / -48 | 4,369 (10%) \|  13 / 16 | 42,330 (5%) \|  -47 / -42 |
|  | 72,515 (64%) \|  -34 / -36 | 41,624 (36%) \|  19 / 22 | 114,139 (3%) \|  -15 / -15 |
| Total  CSV  Non-CSV |  | | |
|  | 718,225 (87%) \|  -50 / -47 | 107,468 (13%) \|  14 / 17 | 825,693 \|  -44 / -39 |
|  | 2,117,406 (55%) \|  -32 / -34 | 1,707,842 (45%) \|  22 / 24 | 3,825,248 \|  -6 / -8 |

**Supplementary Table 2: Distribution of variants and their predicted effects by human population frequency.** Cells in ‘Neutral’ & ‘Effect’ rows list variant fractions of ‘All’ row.

|  | **CSV** | | **non-CSV** | | **Combined (total)** | |
| --- | --- | --- | --- | --- | --- | --- |
|  | **Rare**  (% of all) | **Common**  (% of all) | **Rare**  (% of all) | **Common**  (% of all) | **Rare**  (% of all) | **Common**  (% of all) |
| Neutral | 37,410  (90%) | 551  (80%) | 72,107  (64%) | 408  (60%) | 109,517  (71%) | 959  (70%) |
| Effect | 4,229 (10%) | 140 (20%) | 41,349  (36%) | 275  (40%) | 45,578  (29%) | 415  (30%) |
| All | 41,639 | 691 | 113,456 | 683 | 155,095 | 1,374 |

**Supplementary Table 3: funtrp class prediction fractions for variant sets**

|  | Synthetic | | Singleton | | Observed | |
| --- | --- | --- | --- | --- | --- | --- |
|  | CSV | Non-CSV | CSV | Non-CSV | CSV | Non-CSV |
| Neutrals | 61% | 47% | 61% | 50% | 62% | 53% |
| Rheostats | 30% | 36% | 31% | 33% | 30% | 31% |
| Toggles | 9% | 18% | 8% | 17% | 8% | 16% |
